# Prediction of inpatient pressure ulcers based on routine healthcare data using machine learning methodology

**DOI:** 10.1038/s41598-022-09050-x

**Published:** 2022-03-23

**Authors:** Felix Walther, Luise Heinrich, Jochen Schmitt, Maria Eberlein-Gonska, Martin Roessler

**Affiliations:** 1 Center for Evidence-Based Healthcare, Medical Faculty Carl Gustav Carus, TU Dresden, Fetscherstraße 74, 01307 Dresden, Germany; 2 Quality and Medical Risk Management, University Hospital Carl Gustav Carus, Fetscherstraße 74, 01307 Dresden, Germany

**Keywords:** Disease prevention, Health services, Health care, Risk factors

## Abstract

Despite the relevance of pressure ulcers (PU) in inpatient care, the predictive power and role of care-related risk factors (e.g. anesthesia) remain unclear. We investigated the predictability of PU incidence and its association with multiple care variables. We included all somatic cases between 2014 and 2018 with length of stay ≥ 2d in a German university hospital. For regression analyses and prediction we used Bayesian Additive Regression Trees (BART) as nonparametric modeling approach. To assess predictive accuracy, we compared BART, random forest, logistic regression (LR) and least absolute shrinkage and selection operator (LASSO) using area under the curve (AUC), confusion matrices and multiple indicators of predictive performance (e.g. sensitivity, specificity, F1, positive/ negative predictive value) in the full dataset and subgroups. Analysing 149,006 cases revealed high predictive variable importance and associations between incident PU and ventilation, age, anesthesia (≥ 1 h) and number of care-involved wards. Despite high AUCs (range 0.89–0.90), many false negative predictions led to low sensitivity (range 0.04–0.10). Ventilation, age, anesthesia and number of care-involved wards were associated with incident PU. Using anesthesia as a proxy for immobility, an hourly repositioning is indicated. The low sensitivity indicates major challenges for correctly predicting PU based on routine data.

## Introduction

## Introduction

Pressure ulcers (PU) are serious adverse events in inpatient care. Constant pressure caused by limited mobility due to e.g. ventilation, anesthesia or other severe physical or mental impairments leads to reduced blood perfusion of tissues. The ischemia leads to hypoxia of the tissue. The arising toxic metabolites lead to irreversible damage of nerve cells and, in most severe cases, to necrosis. In addition, age and age-related comorbidities like type 2 diabetes, dementia, obesity or incontinence, severely increase the risk of PU^1^. Due to the fact that this adverse event can be prevented in the majority of cases PUs are a well-established patient safety outcome and content of inpatient quality assurance in multiple countries^2^. Depending on the legislation, inpatient care providers need to report this patient safety outcome on the basis of uniformly defined and standardized data sets. Consequently, large routine data sets are evaluated by the responsible authority using statistical methods like logistic regression. For benchmarking purposes, results are usually expressed as indicators statistically adjusting for patient age, comorbidities (e.g., type 2 diabetes, infections, immobility) or intensive care with ventilation^2^. In the event of outliers corrective measures are triggered by the responsible authority. Previous works suggested that care-related risk factors like reason for admission (e.g. emergency vs. referral), (length of) performed surgery, intensive care or wards involved in care play an important role^3–7^. These risk factors also serve as possible proxies for the acuity of a medical case. As the high prevalence of different comorbidities like diabetes mellitus leads to a relatively large high-risk population an early identification of patients at risk is crucial for an early prevention of PU^8^. Furthermore, risk factors can interact and thus significantly increase a patient's risk of developing a PU. In this case, simple approaches to statistical adjustment are inappropriate, particularly regarding prospective prediction of PU.

One approach of handling complex interactions is stratification into small and homogeneous patient groups, which has the disadvantage of low statistical power and precision^9^. An another approach is the use of non-parametric statistical methods that facilitate data-driven detection of complex interactions between risk factors and flexible investigation of relationships with the outcome. One of these approaches is the machine learning method Bayesian Additive Regression Trees (BART). Similar to all nonparametric machine learning methods, BART has the advantage that the researcher does not have to specify the functional form of the predictive relationship between outcome and risk factors. Instead, these relationships are learned from the data and may include complex interactions between risk factors and highly nonlinear and non-monotonic relationships between risk factors and outcome. At the same time, BART is a fully Bayesian approach and allows for statistical inference, e.g. in terms of derivation of credible intervals^10^.

Based on an appropriate risk identification, repositioning is a widely established and guideline-recommended pressure ulcer prevention strategy^11,12^. Despite widespread acceptance in clinical practice the determination of an evidence based time interval for repositioning is still missing. A recent Cochrane review systematically reviewed, critically appraised and summarized the randomized evidence concerning this question. The review showed no differences between the widely practiced two hour- or longer repositioning frequencies. However, the included RCTs had small sample sizes and were of poor study quality^13,14^.

BART as a non-parametric statistical method is able to handle continuous variables without assuming linearity in the predictor term as is inherent to logistic regression^10,15^. Therefore BART affords the user the opportunity to model the incidence of pressure ulcers related to the continuous length of anesthesia appropriately—without assuming linearity.

In summary, two aims have been identified for the analysis of PU using BART as a machine learning approach in a large routine data set of a tertiary care provider:

1. To explore *relationships* between incidence of PU and
  (length of) anesthesia,
  wards involved in care and
  admission reasons (emergency, transfer from another hospital)
  intensive care treatment (with/ without ventilation),
  adjusting for age, sex and comorbidities.
2. To examine *predictability* of pressure ulcers using BART based on routine data

## Results

Overall, 149,006 cases were included for analysis (51.5% male, median age 64 years, interquartile range 48–76 years). Incident pressure ulcers were documented in 4,663 cases (3.1%). With respect to the test year 2018, 901 incident pressure ulcers (3.0%) in 29,338 hospital cases were documented (Table 1). Referring to admission context, around one third (35.8%) of the included cases were admitted as emergency case and 3.5% were transferred from other hospitals. Around the half of the cases (49.7%) included surgery and full anesthesia. More than 50% of the analyzed cases were treated on one ward. One fifth (19.6%) of the cases analyzed received intensive care with (4.1%) or without (15.5%) ventilation.

**Table 1:** Patient and care characteristics of 149,006 analyzed cases between 2014 and 2018.

Outcome/variable	Overall		Training data (2014–2017)		Test data (2018)	
	n/median	%/Q1; Q3	n/median	%/Q1; Q3	n/median	%/Q1; Q3
**Incident pressure ulcer**						
Yes	4,663	(3.1%)	3,757	(3.1%)	906	(3.1%)
No	144,343	(96.9%)	115,911	(96.9%)	28,432	(96.9%)
**Age**						
Median	64	(48;76)	64	(47;75)	64	(49;77)
**Male sex**						
Yes	76,774	(51.5%)	61,540	(51.4%)	15,234	(51.9%)
No	72,232	(48.5%)	58,128	(48.6%)	14,104	(48.1%)
**Diabetes mellitus type 2**						
Yes	26,893	(18%)	21,402	(17.9%)	5,491	(18.7%)
No	122,113	(82%)	98,266	(82.1%)	23,847	(81.3%)
**BMI ≥ 40**						
Yes	1,603	(1.1%)	1,200	(1.0%)	403	(1.4%)
No	147,403	(98.9%)	118,468	(99.0%)	28,935	(98.6%)
**Underweight and/or malnutrition**						
Yes	1,069	(0.7%)	813	(0.7%)	256	(0.9%)
No	147,937	(99.3%)	118,855	(99.3%)	29,082	(99.1%)
**Dementia and/or vigilance disturbance**						
Yes	4,167	(2.8%)	3,200	(2.7%)	967	(3.3%)
No	144,839	(97.2%)	116,468	(97.3%)	28,371	(96.7%)
**Infections**						
Yes	8,866	(6%)	6,914	(5.8%)	1,952	(6.7%)
No	140,140	(94%)	112,754	(94.2%)	27,386	(93.3%)
Other severe diseases*						
Yes	32,988	(22.1%)	25,795	(21.6%)	7,193	(24.5%)
No	116,018	(77.9%)	93,873	(78.4%)	22,145	(75.5%)
**Mobility**						
Yes	10,016	(6.7%)	7,716	(6.4%)	2,300	(7.8%)
No	138,990	(93.3%)	111,952	(93.6%)	27,038	(92.2%)
**Incontinence**						
Yes	13,287	(8.9%)	10,558	(8.8%)	2,729	(9.3%)
No	135,719	(91.1%)	109,110	(91.2%)	26,609	(90.7%)
**Admission: emergency case**						
Yes	53,418	(35.8%)	42,593	(35.6%)	10,825	(36.9%)
No	95,588	(64.2%)	77,075	(64.4%)	18,513	(63.1%)
**Admission: transfer from another hospital**						
Yes	5,275	(3.5%)	4,195	(3.5%)	1,080	(3.7%)
No	143,731	(96.5%)	115,473	(96.5%)	28,258	(96.3%)
**Anesthesia**						
Yes	74,037	(49.7%)	59,049	(49.3%)	14,988	(51.1%)
No	74,969	(50.3%)	60,619	(50.7%)	14,350	(48.9%)
**Length of anesthesia (minutes)**						
Median	142	(87;214)	0	(0;140)	37	(0;150)
**Wards involved in care**						
Median	1	(1;2)	1	(1;2)	1	(1;2)
**Intensive care with ventilation**						
Yes	6,106	(4.1%)	4,854	(4.1%)	1,252	(4.3%)
No	142,900	(95.9%)	114,814	(95.9%)	28,086	(95.7%)
**Intensive care without ventilation**						
Yes	23,041	(15.5%)	18,636	(15.6%)	4,405	(15%)
No	125,965	(84.5%)	101,032	(84.4%)	24,933	(85%)

*Candidiasis (B37.1, B37.7) anemia (D50-D53, D61-D64, D72.8), liver diseases (K70, K72, K74), renal diseases (N17, N18.4, N18.5, N99.0, Z99.2), ascites (R18), anuria (R34), diabetic polyneuropathy (G63.2), oedema (R60), abnormality of albumin (R77), hospital acquired pneumonia (U69.00!).

### Variable importance derived from BART model

**Figure 1:**
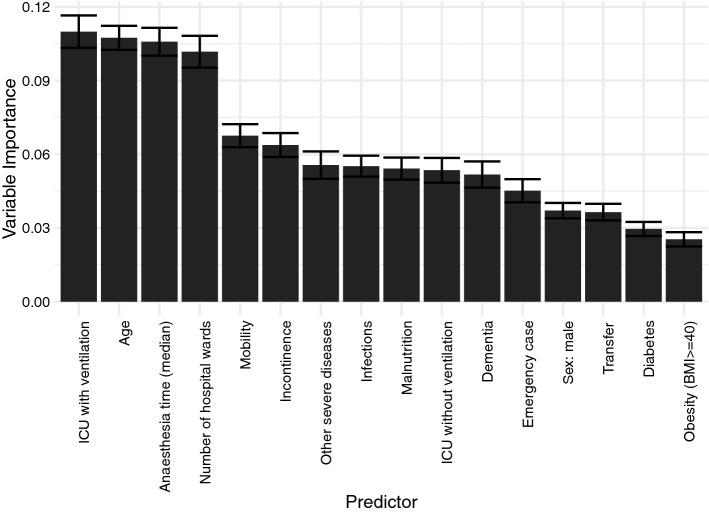
Variable importance. The variable importance shows which variable (predictor) was most predictive for incident pressure ulcer from the highest (ICU with ventilation) to the lowest (BMI ≥ 40) predictive power.

According to the results of a tenfold cross validation, a BART model with 50 trees yielded the best predictive performance and, thus, was chosen for analysis. Variable importances (Fig. 1) derived from the fitted BART model were highest for.

- ICU with ventilation (0.109)
- Age (0.107)
- length of anesthesia (0.105)
- the number of wards involved in care of the patient (0.101)

The prognosis of incident PU is particularly influenced by these 4 variables. If a higher age or ICU with ventilation is given, then the model tends to predict an incidental PU. The most important comorbidity variables were mobility (0.067) and incontinence (0.063).

### Regression analysis of care-related risk factors on the predicted probability of pressure ulcers

**Figure 2:**
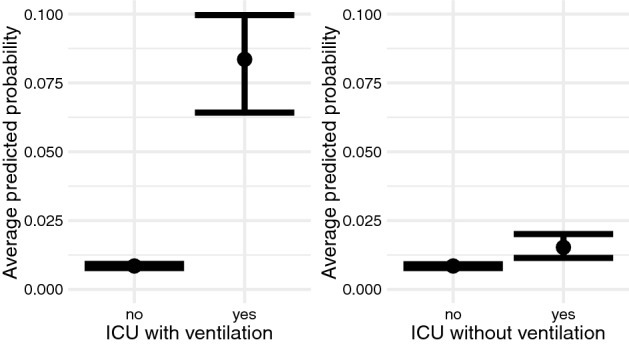
Associations between intensive care and incident pressure ulcer. Average predicted probability between non-intensive care, intensive care with ventilation, intensive care without ventilation and the incidence of pressure ulcers at a 95% credibility interval.

According to the estimated partial dependence, the average predicted probability of pressure ulcers for intensive care with ventilation was about 8 times (7.5 percentage points) higher than for cases with neither intensive care nor ventilation. Comparing intensive care with and without ventilation, the average predicted probability of incident PU was 4 times higher (6.8 percentage points) in cases treated with ventilation (Fig. 2).

**Figure 3:**
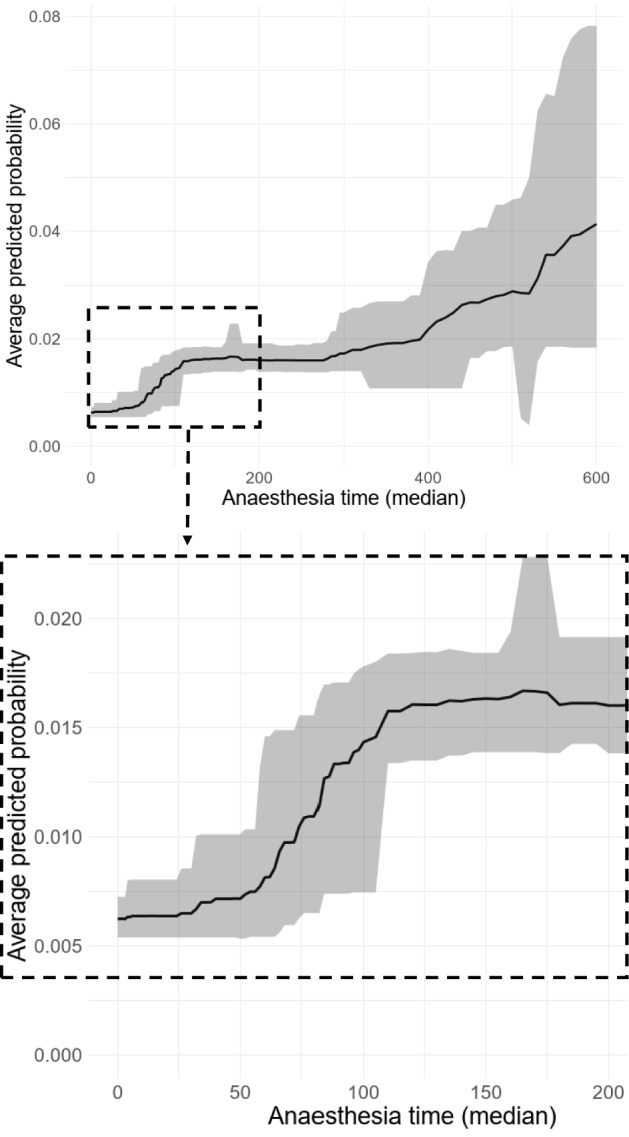
Associations between length of anesthesia and incident pressure ulcer. Average predicted probability between length of anesthesia and the incidence of pressure ulcers at a 95% credibility interval.

Anesthesia in general was associated with an increased risk of PU (Fig. 3). A monotonous increase was observed between 50 and 120 min of anesthesia. In this timeframe, the average predicted probability of incident pressure ulcer doubled. Between 120 and 240 min of anesthesia, the average predicted probability remained stable and increased after 240 min of anesthesia with a broadening credible interval.

**Figure 4:**
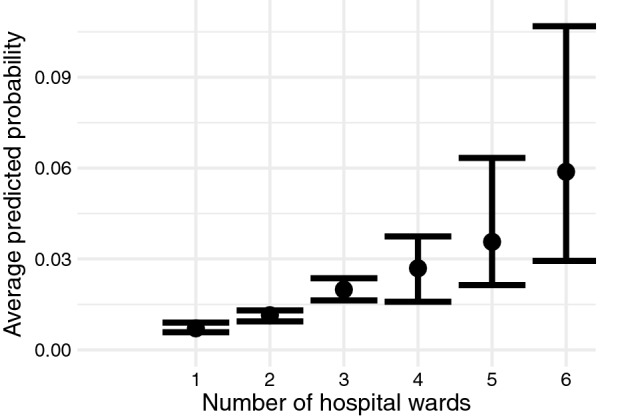
Associations between number of wards involved into care and incident pressure ulcer. Average predicted probability between the number of wards involved in care and the incidence of pressure ulcers at a 95% credibility interval.

The average predicted probability of incident pressure ulcer was higher, when more than one hospital ward was involved in care (Fig. 4).

**Figure 5:**
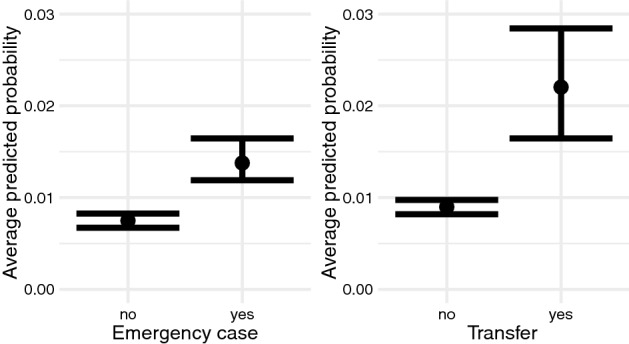
Associations between emergency admissions, transfers from another hospital and incident pressure ulcer. Average predicted probability between non-urgent admissions, emergency admissions, transfers from another hospital and the incidence of pressure ulcers at a 95% credibility interval.

The remaining care variables such as admission as emergency case or transfer from another hospital were related to a higher average predicted probability of incident PU compared to referral admissions (Fig. 5).

The average predicted probability for incident PU was 1.5 times higher (1.45 percentage points) in cases transferred from other hospitals than in referral admissions.

While the average predicted probability of incident PU remained constant for patients aged 19–35, the average predicted risk of incident PU monotonously increased for patients aged 35–86 (Supplement S1). Reviewing age in more detail, the predicted probability of incident PU increased constantly by a total of 0.10 percentage points between the age of 35 and 50. Between the age of about 50 and 90, the average predicted probability triples (1.9 percentage points) with a broadening credible interval. Please refer to Supplement S2 and Supplement S3 for the partial dependence plots of male sex and comorbidities.

To illustrate potential patient-specific differences in the predicted risk of pressure ulcers, we considered five examples (Table 2). As most possible risk factors were absent, examples 1 and 2 showed a predicted probability of almost 0%. With longer anesthesia, more comorbidities and intensive care without ventilation, examples 3 and 4 showed increased risks of 6% and 11%, respectively. Due to the presence of multiple risk factors like higher age (70 years), long anesthesia (150 min) and intensive care with ventilation, example 5 showed a high predicted risk of 42% for incident pressure ulcer.

**Table 2:** Predicting incident pressure ulcer based on 5 examples of different risk factors and age.

	Example 1	Example 2	Example 3	Example 4	Example 5
Age	20	35	45	50	70
Male sex	Yes	No	Yes	No	Yes
Diabetes	No	No	Yes	Yes	No
BMI ≥ 40	No	No	No	No	No
Underweight and malnutrition	No	No	No	No	Yes
Dementia and vigilance disturbance	No	No	No	No	Yes
Infections	No	No	No	Yes	No
Other severe diseases	No	No	Yes	Yes	No
Mobility	No	No	Yes	No	Yes
Incontinence	No	No	No	No	Yes
Admission: emergency case	No	No	No	Yes	No
Admission: transfer from another hospital	No	No	No	No	Yes
Length of anesthesia	0	60	100	120	150
Wards involved in care	2	1	1	2	4
Intensive care with ventilation	No	No	No	No	Yes
Intensive care without ventilation	No	No	Yes	Yes	No
Predicted probability					
(Low CI-High CI)	0,00 (0,00–0,00)	0,00 (0,00–0,00)	0,06 (0,02–0,1)	0,11 (0,05–0,2)	0,42 (0,15–0,72)

*CI* credible interval.

### Predictive performance measures

The area under the curve was 0.9 for BART and 0.89 for LASSO, LR and random forest (Supplement S4).

Applied to the whole dataset, between 40 (4.4%) and 80 (8.8%) true positive PU cases in 2018 could be predicted with the models trained on data from 2014 to 2017 (Supplement S5). Between 28,294 (96.4%) and 28,369 (99.8%) cases were correctly predicted as true negative. False negative PU predictions ranged between 816 (logistic regression) and 866 (LASSO) and false positive PU predictions between 39 (LASSO) and 138 (LR). Evaluation metrics like F1-score (range 0.08–0.16), balanced accuracy (range 0.52–0.55) and positive predictive value (range 0.39–0.58) varied between the different models (Supplement S6). The sensitivity of the prediction for the full dataset ranged between 0.04 (LASSO) and 0.10 (LR). When predicting for cases receiving intensive care, ventilation and anesthesia, evaluation scores like F1 (range 0.10–0.33), positive predictive value (range 0.40–0.59), balanced accuracy (range 0.53–0.59) and sensitivity (range 0.10–0.24) increased. In case of true negative predictions, negative predictive value (0.97) and specificity (1.00) remained stable in the full dataset for all four models. In low-risk groups (no intensive care, no surgery, no anesthesia) specificity also remained stable (1.00) and negative predictive value varied (0.98–0.99).

The prediction of severity in incident PU revealed the lowest sensitivities for less severe PU grades (Grade 1–2: range 0.02–0.09). Sensitivity increased when predicting more severe PU (Grade 4: range 0.17–0.21, Supplement S7).

## Discussion

This large observational study presents several important new findings that are relevant for inpatient care of pressure ulcers.

*First*, critical length of anesthesia has not been determined with a visible threshold before. According to our results, the average predicted probability of incident pressure ulcers begins to increase at 50 min of anesthesia. Subsequently, the probability of incident pressure ulcers steeply increases until reaching a plateau between 120 and 240 min before increasing again. Especially the plateau of incident pressure ulcer risk between 120 and 240 min of limited/ not provided repositioning is in line with the RCTs published up to date and may reflect preventive measures^12^. Considering the whole process of anesthesia with induction, excitement stage, surgical anesthesia and awakening, even short surgeries with their steep increase of incident pressure ulcers within one hour and despite the possible use of preventive interventions can be interpreted as a risk factor despite the possible use of preventive interventions. If this situation is applied to the restricted mobility of many inpatients, this would result in an hourly, timely tightened rather than delayed repositioning in general. The recommendation for a tightened repositioning beginning at approximately 50 min of immobility puts our results in contrast to RCTs published up to date which have not even considered such short repositioning intervals^13,14^. Probably due to small sample sizes/ underpowered comparisons^13^, some RCTs suggest longer repositioning intervals of three to four hours of immobility compared to a control group of 2-h repositioning intervals^14,16,17^. Repositioning every two hours is common in clinical practice but not based on reliable evidence^13^. However, it is also necessary to consider the burden to the patient (e.g., sleep disturbances) and staff (e.g., back pain due to manual handling activities) which might result from hourly repositioning^18–20^. As we used observational data from a broad inpatient sample we strongly recommend a (randomized) controlled design with a sufficiently large sample size to provide confirmatory evidence on the hourly repositioning intervals indicated by this analysis.

*Second,* the high AUCs of 0.89 LR, LASSO and random forest and 0.90 for BART suggest a strong predictive performance. However, less than 10% of cases with pressure ulcers in 2018 could be predicted, and lead to very low sensitivity scores in both full dataset and subgroups (low risk, high risk, grades of incident PU). This suboptimal performance of the prediction models could be explained by multiple reasons.

- The high class imbalance between incidental PU (3.1%) and non-PU (96.9%) might weaken the performance of ROC—analyses regardless of the model chosen^21^.
- The high class imbalance between incidental and non-PU also might explain the high specificity and negative predictive value in all models and subgroups included.
- The development of PU has multifactorial causes. For example, our regression model also indicates intensive care with ventilation as a risk factor in addition to age, anesthesia time, comorbidities, incontinence and so on. Some relevant risk factors (e.g. state of consciousness, pain perception, body temperature, medication)^1^ may not have been included in our data
- Not every risk factor can be coded well in its severity in secondary data and ICD-10- Codes^22^. For example, an infection may be a local infection or it may have already spread to the bloodstream and organs. Limited mobility might range from walking disability, to the need for a wheelchair or to complete bed confinement. These aspects are not captured by our data.
- The heterogeneity of the underlying risk factors also could weaken the predictive performance.

Given our statistical models, higher sensitivity would be possible but would come at the cost of specificity. This generally highlights the importance of further research on additional strong predictors of pressure ulcers. However, despite the modest predictive performance of the model, relationships between risk factors and the predicted probability of incident pressure ulcers could be estimated with relatively high precision due to the large sample size.

*Third*, identification of age and intensive care with ventilation as crucial risk factors are in line with the literature^1,7^. Comorbidities, male sex or admission reasons on the other hand did neither reveal high variable importance nor high average predicted probabilities for incident pressure ulcers in a broad, medical complex (e.g. intensive care) and older age sample.

This study analyzed a large sample with a broad range of medical indications as is common in tertiary care facilities. Statistically, flexible predictive analysis using BART as a nonparametric machine learning technique allowed us to handle continuous variables like length of anesthesia or age without presuming specific functional forms of their relationships with the risk of pressure ulcers. The use of referenced and predefined risk factors aiming at specific adjustment and the use of a machine learning approach like BART enabled a tailored and literature-based model. Routine data in general often face a lack of granularity with respect to complete coding and missing time references^22^. In addition, routine data do not always include information on which diagnoses were already present on admission and which were not^23,24^. These challenges could be solved due to the use of multiple data sources to acquire a complete and longitudinal data set. Based on our results and the clear visible thresholds, we are able to derive actionable implications. The monocentric setting can be seen as a limitation with respect to the generalizability of provider-specific structures and processes^25^. However, the setting of an university hospital with its organizationally independent and large clinics, the data completeness and variety underlines the (necessary) medical plurality of this analysis. Due to data protection issues, patients admitted more than once could not be identified, which implies that some patients may have entered the analysis as multiple hospital cases. This large routine data set inhibited a detailed analysis of the administered, and often multimodal, preventive interventions. In general, the use of observational data does not support causal interpretation of results. The routine data collected did not include explicit repositioning time protocols which lead us to use the length of anesthesia as a proxy for limited mobility. This definition might be biased by selection and strongly highlights the need for controlled designs to validate our results.

In addition to well-known risk factors like age, comorbidities and intensive care treatment, our analysis indicates anesthesia and repositioning intervals longer than 50 min as relevant predictors of pressure ulcers. As our results are based on observational data and repositioning needs to consider patients and staff burden, a randomized controlled trial in a large sample would be valuable.

## Methods

We conducted a mono-centered cross-sectional study at a tertiary care facility. This study has been carried out in accordance to STROBE as general guideline for observational studies^26^ and in particular STROSA for studies analyzing secondary data^27^.

### Population

We included all adult (≥ 19 years) cases admitted and discharged between 2014 and 2018 in the University Hospital Carl Gustav Carus, Dresden. We excluded children/ adolescents, cases with prevalent PU, psychiatric treatment and length of stay < 2 days.

### Outcomes and covariates

The outcome/dependent variable was case-specific incident PU. To correctly identify prevalent and incident PU, a consistent assessment beginning at admission is essential. Especially in nursing home residents, it is not always clear whether a pressure ulcer was already present on admission. Our in-house standard requires a pressure ulcer assessment for high risk cases (internistic treatment, intensive care and surgery) within 24 h from admission. Every PU detected within this timeframe has been marked as prevalent and excluded from our analysis.

We grouped the independent variables into case- and care-related characteristics.

Case-related characteristics include age, (male) sex and comorbidities. To define comorbidities (based on ICD-10) appropriately, we followed the German inpatient quality assurance program. The German inpatient quality assurance indicator for PU adjusts for Diabetes mellitus type 2, BMI ≥ 40, underweight and/ or malnutrition, dementia and/ or vigilance disturbance, infections, other severe diseases, mobility and incontinence. The ICD-10-based definitions are provided in Supplement S8^28^.

Care-related characteristics include admission reasons (emergency case, transfer from another hospital), (length of) surgical anesthesia, number of wards involved in care and intensive care with or without ventilation.

We did not include the Braden score as predictor in the models since it was used for preventive PU screening in the hospital. This implies that likely cases of PU indicated by the Braden score may have been prevented and do not occur in our data. Accordingly, estimating relationships between observed PUs and the Braden score would induce misleading results. Some literature also adds length of hospital stay in risk-adjusted analyses for pressure ulcer on the one hand^29,30^. On the other hand, several studies showed that pressure ulcers extend the length of hospital stay^31–33^. This feedback effect causes endogeneity of length of hospital stay as a predictor of pressure ulcer and could seriously bias the results of our risk factor analysis. Therefore, we decided not to consider length of stay as part of the main analysis. However, we included length of stay as a predictor for sensitivity analysis (Supplement S9). In the main analysis, case complexity was captured by a wide set of variables such as comorbidities, anesthesia, reason for admission, intensive care treatment and ventilation.

### Data sources

We used four data sources:

I. internally standardized and routinely collected PU screening for the detection of incident PU,
II. legally (§21 Krankenhausentgeltgesetz) required and prespecified accounting data for age, sex, comorbidities, intensive care treatment, ventilation and admission reasons,
III. case-based surgery protocols for length of surgical anesthesia (induction to awakening)
IV. case-based ward stays for the number of involved hospital wards per case

### Study participation, privacy, and ethics

We analyzed pseudonymized routine datasets in a mono-centered setting. If reasonably justified, the legislation of the federal state of Saxony (§35(1–3) "Sächsisches Krankenhausgesetz") does not require individual consent for large pseudonymized and mono-centric routine datasets. The legal justification in the federal state of Saxony is based on the principle of in-house research by the specific providers. We have integrated these data privacy relevant conditions and justifications into our study protocol. The Institutional Review Board (IRB00001473 and IORG0001076) of the Medical Faculty of the TU Dresden reviewed and approved the study protocol.

### Patient and public involvement

It was not appropriate to involve patients or the public in the design, or conduct, or reporting, or dissemination plans of our research. This is a non-interventional cross-sectional analysis based on observational data, predefined outcomes and covariates.

### Statistical methods

Descriptive statistics in case of categorical variables were provided as absolute and relative frequencies. Continuous variables were described by the median and the 1st and 3rd quartile. We used Bayesian Additive Regression Trees (BART) to predict pressure ulcers and estimate predictive relationships between pressure ulcers and risk factors^10^. Generally, BART is based on regression trees, which may be used when associations between independent and dependent variables cannot be described linearly. The advantage of regression trees over, e.g., logistic regression is the ability to handle non-logistic associations and interactions. Regression trees build homogeneous groups to identify relationships between the outcome and covariates. At a certain degree of heterogeneity in the groups, the groups are separated to achieve higher homogeneity (splitting). BART combines multiple trees in a “sum-of-trees” model, which facilitates more accurate and stable out-of-sample predictions than single regression trees. This ability led us to prospectively predict incidences of PU in addition to associations between dependent and independent variables. In this regard, it is noteworthy that a high/low predictive power of a model does not necessarily imply accurate/inaccurate estimation of relationships between outcome and covariates^34^.

We used data from 2014 to 2017 to fit the BART model. The number of trees (50, 75, 100) served as tuning parameter in tenfold cross validation. We assessed the predictive performance of the selected model based on a confusion matrix and area under the curve (AUC) using data from 2018. An AUC of 0.5 suggests no discrimination (i.e., ability to predict cases with and without incident PU), 0.7 to 0.8 is considered acceptable, 0.8 to 0.9 is considered excellent, and more than 0.9 is considered outstanding^35^. In addition to confusion matrices, we analysed performance indicators sensitivity, specificity, positive predictive value, negative predictive value, precision, recall, F1, prevalence, detection rate, detection prevalence, balanced accuracy (in case of high class imbalance) and accuracy. Subgroup analyses were performed for the full dataset, intensive care (yes/no) anesthesia (yes/no), ventilation (yes/no) and the different grades of PU. To assess the predictive performance of specific risk factors, we calculated variable importance as the proportion of times each risk factor was chosen for a splitting rule, i.e. to define a node in the sum-of-trees model. We calculated partial dependences to explore the influence of risk factors (e.g. age) on the predicted probability of pressure ulcers. We used 95%-credible intervals to assess the precision of partial dependence estimates. Statistical analysis was conducted using R 3.6.3 and the package bartMachine^36^. With respect to methodological rigor, the accuracy of BART predictions was compared with those based on multiple logistic regression, random forest, and LASSO (see Supplement S10 for a more detailed description).

## Ethics approval

The Institutional Review Board (IRB00001473 and IORG0001076) of the Medical Faculty of the TU Dresden reviewed and approved the study protocol.

## Supplementary Information

Supplementary Information S5–S10.

Supplementary Information 1.

Supplementary Information 2.

Supplementary Information 3.

Supplementary Information 4.

Supplementary Information 9.
