# Supplementary material for: Prediction of inpatient pressure ulcers based on routine healthcare data using machine learning methodology
Source: Sci Rep. 2022 Mar 23;12:5044. doi: 10.1038/s41598-022-09050-x (PMC8943147; doi:10.1038/s41598-022-09050-x)
Supplement: Supplementary file 1 — Supplementary Information S5–S10. [file 41598_2022_9050_MOESM1_ESM.docx]

**Supplements**

**S1 - Average predicted probability of incident pressure ulcers related to age**

**S2 - Average predicted probability of incident pressure ulcers related to male sex**

**S3 - Average predicted probability of incident pressure ulcers related to comorbidities**

**S4 - ROC curve of logistic regression, random forest, LASSO and BART**

**S5 - Confusion Matrix comparing BART, LASSO, logistic regression and random forest in full dataset and subgroups**

|  | **Full dataset** | | **Intensive care** | | **No intensive care** | | **Ventilation** | | **No ventilation** | | **Anesthesia** | | **No anesthesia** | |
| --- | --- | --- | --- | --- | --- | --- | --- | --- | --- | --- | --- | --- | --- | --- |
|  | actual yes | actual no | actual yes | actual no | actual yes | actual no | actual yes | actual no | actual yes | actual no | actual yes | actual no | actual yes | actual no |
| *BART* |  |  |  |  |  |  |  |  |  |  |  |  |  |  |
| Predicted yes | 80 | 63 | 79 | 62 | 1 | 1 | 74 | 52 | 6 | 11 | 79 | 59 | 1 | 4 |
| Predicted no | 826 | 28369 | 512 | 5004 | 314 | 23365 | 246 | 880 | 580 | 27489 | 526 | 14324 | 300 | 14045 |
| *LASSO* |  |  |  |  |  |  |  |  |  |  |  |  |  |  |
| Predicted yes | 40 | 39 | 39 | 39 | 1 | 0 | 39 | 35 | 1 | 4 | 35 | 29 | 5 | 10 |
| Predicted no | 866 | 28393 | 552 | 5027 | 314 | 23366 | 281 | 897 | 585 | 27496 | 570 | 14354 | 296 | 14039 |
| *Logistic regression* |  |  |  |  |  |  |  |  |  |  |  |  |  |  |
| Predicted yes | 90 | 138 | 89 | 132 | 1 | 6 | 77 | 106 | 13 | 32 | 77 | 91 | 13 | 47 |
| Predicted no | 816 | 28294 | 502 | 4934 | 314 | 23360 | 243 | 826 | 573 | 27468 | 528 | 14292 | 288 | 14002 |
| *Random forest* |  |  |  |  |  |  |  |  |  |  |  |  |  |  |
| Predicted yes | 61 | 44 | 61 | 44 | 0 | 0 | 58 | 41 | 3 | 3 | 60 | 41 | 1 | 3 |
| Predicted no | 845 | 28388 | 530 | 5022 | 315 | 23366 | 262 | 891 | 583 | 27497 | 545 | 14342 | 300 | 14046 |

**S6 - Predictive performance measures for BART, logistic regression, LASSO and random forest**

|  | **full dataset** | **intensive care** |  | **ventilation** |  | **anesthesia** |  |
| --- | --- | --- | --- | --- | --- | --- | --- |
|  |  | yes | no | yes | no | yes | no |
| *BART* |  |  |  |  |  |  |  |
| Sensitivity | 0.09 | 0.13 | 0.00 | 0.23 | 0.01 | 0.13 | 0.00 |
| Specificity | 1.00 | 0.99 | 1.00 | 0.94 | 1.00 | 1.00 | 1.00 |
| Positive predictive value | 0.56 | 0.56 | 0.50 | 0.59 | 0.35 | 0.57 | 0.20 |
| Negative predictive value | 0.97 | 0.91 | 0.99 | 0.78 | 0.98 | 0.96 | 0.98 |
| Precision | 0.56 | 0.56 | 0.50 | 0.59 | 0.35 | 0.57 | 0.20 |
| Recall | 0.09 | 0.13 | 0.00 | 0.23 | 0.01 | 0.13 | 0.00 |
| F1 | 0.15 | 0.22 | 0.01 | 0.33 | 0.02 | 0.21 | 0.01 |
| Prevalence | 0.03 | 0.10 | 0.01 | 0.26 | 0.02 | 0.04 | 0.02 |
| Detection rate | 0.00 | 0.01 | 0.00 | 0.06 | 0.00 | 0.01 | 0.00 |
| Detection prevalence | 0.00 | 0.02 | 0.00 | 0.10 | 0.00 | 0.01 | 0.00 |
| Balanced accuracy | 0.54 | 0.56 | 0.50 | 0.59 | 0.50 | 0.56 | 0.50 |
| Accuracy | 0.97 | 0.90 | 0.99 | 0.76 | 0.98 | 0.96 | 0.98 |
| *LASSO* |  |  |  |  |  |  |  |
| Sensitivity | 0.04 | 0.07 | 0.00 | 0.12 | 0.00 | 0.06 | 0.02 |
| Specificity | 1.00 | 0.99 | 1.00 | 0.96 | 1.00 | 1.00 | 1.00 |
| Positive predictive value | 0.51 | 0.50 | 1.00 | 0.53 | 0.20 | 0.55 | 0.33 |
| Negative predictive value | 0.97 | 0.90 | 0.99 | 0.76 | 0.98 | 0.96 | 0.98 |
| Precision | 0.51 | 0.50 | 1.00 | 0.53 | 0.20 | 0.55 | 0.33 |
| Recall | 0.04 | 0.07 | 0.00 | 0.12 | 0.00 | 0.06 | 0.02 |
| F1 | 0.08 | 0.12 | 0.01 | 0.20 | 0.00 | 0.10 | 0.03 |
| Prevalence | 0.03 | 0.10 | 0.01 | 0.26 | 0.02 | 0.04 | 0.02 |
| Detection rate | 0.00 | 0.01 | 0.00 | 0.03 | 0.00 | 0.00 | 0.00 |
| Detection prevalence | 0.00 | 0.01 | 0.00 | 0.06 | 0.00 | 0.00 | 0.00 |
| Balanced accuracy | 0.52 | 0.53 | 0.50 | 0.54 | 0.50 | 0.53 | 0.51 |
| Accuracy | 0.97 | 0.90 | 0.99 | 0.75 | 0.98 | 0.96 | 0.98 |
| *Logistic regression* |  |  |  |  |  |  |  |
| Sensitivity | 0.10 | 0.15 | 0.00 | 0.24 | 0.02 | 0.13 | 0.04 |
| Specificity | 1.00 | 0.97 | 1.00 | 0.89 | 1.00 | 0.99 | 1.00 |
| Positive predictive value | 0.39 | 0.40 | 0.14 | 0.42 | 0.29 | 0.46 | 0.22 |
| Negative predictive value | 0.97 | 0.91 | 0.99 | 0.77 | 0.98 | 0.96 | 0.98 |
| Precision | 0.39 | 0.40 | 0.14 | 0.42 | 0.29 | 0.46 | 0.22 |
| Recall | 0.10 | 0.15 | 0.00 | 0.24 | 0.02 | 0.13 | 0.04 |
| F1 | 0.16 | 0.22 | 0.01 | 0.31 | 0.04 | 0.20 | 0.07 |
| Prevalence | 0.03 | 0.10 | 0.01 | 0.26 | 0.02 | 0.04 | 0.02 |
| Detection rate | 0.00 | 0.02 | 0.00 | 0.06 | 0.00 | 0.01 | 0.00 |
| Detection prevalence | 0.01 | 0.04 | 0.00 | 0.15 | 0.00 | 0.01 | 0.00 |
| Balanced accuracy | 0.55 | 0.56 | 0.50 | 0.56 | 0.51 | 0.56 | 0.52 |
| Accuracy | 0.97 | 0.89 | 0.99 | 0.72 | 0.98 | 0.96 | 0.98 |
| *Random forest* |  |  |  |  |  |  |  |
| Sensitivity | 0.07 | 0.10 | 0.00 | 0.18 | 0.01 | 0.10 | 0.00 |
| Specificity | 1.00 | 0.99 | 1.00 | 0.96 | 1.00 | 1.00 | 1.00 |
| Positive predictive value | 0.58 | 0.58 | n/a* | 0.59 | 0.50 | 0.59 | 0.25 |
| Negative predictive value | 0.97 | 0.90 | 0.99 | 0.77 | 0.98 | 0.96 | 0.98 |
| Precision | 0.58 | 0.58 | n/a* | 0.59 | 0.50 | 0.59 | 0.25 |
| Recall | 0.07 | 0.10 | 0.00 | 0.18 | 0.01 | 0.10 | 0.00 |
| F1 | 0.12 | 0.18 | n/a* | 0.28 | 0.01 | 0.17 | 0.01 |
| Prevalence | 0.03 | 0.10 | 0.01 | 0.26 | 0.02 | 0.04 | 0.02 |
| Detection rate | 0.00 | 0.01 | 0.00 | 0.05 | 0.00 | 0.00 | 0.00 |
| Detection prevalence | 0.00 | 0.02 | 0.00 | 0.08 | 0.00 | 0.01 | 0.00 |
| Balanced accuracy | 0.53 | 0.55 | 0.50 | 0.57 | 0.50 | 0.55 | 0.50 |
| Accuracy | 0.97 | 0.90 | 0.99 | 0.76 | 0.98 | 0.96 | 0.98 |

Note: n/a*: Random forest did not predict a pressure ulcer for this group. Therefore, positive predictive value, precision and F1-score cannot be calculated.

**S7 - Sensitivity of BART applied for most severe grades of incidental PU**

|  | **Grade 1** | **Grade 2** | **Grade 3** | **Grade 4** |
| --- | --- | --- | --- | --- |
| *BART* | 0.05 | 0.07 | 0.12 | 0.19 |
| *LASSO* | 0.03 | 0.03 | 0.05 | 0.14 |
| *Logistic regression* | 0.06 | 0.09 | 0.12 | 0.21 |
| *Random forest* | 0.02 | 0.05 | 0.11 | 0.17 |
| n | 174 | 437 | 217 | 78 |

**S8 - Definition of comorbidities included based on ICD-10 German Modification**

| BMI≥40 | E66.02* - Obesity due to overconsumption of calories: obesity grade III (WHO) in patients 18 years and older.  E66.12* - Drug-induced obesity: obesity grade III (WHO) in patients 18 years and older.  E66.22* - Excessive obesity with alveolar hypoventilation: obesity grade III (WHO) in patients 18 years and older.  E66.82* - Other obesity: obesity grade III (WHO) in patients 18 years and older.  E66.92* - Obesity, unspecified: Obesity grade III (WHO) in patients 18 years and older. |
| --- | --- |
| Diabetes | E10 - Type 1 diabetes mellitus  E11 - Type 2 diabetes mellitus  E13 - Other specified diabetes mellitus |
| Underweight and malnutrition | E41 - Nutritional marasmus  E43 - Unspecified severe protein-energy malnutrition  E44 - Protein-energy malnutrition of moderate and mild degree  E46 - Unspecified protein-energy malnutrition  R64 - Cachexia |
| Dementia and vigilance disturbance | F00 - Dementia in Alzheimer disease  F01 - Vascular dementia  F02 - Dementia in other diseases classified elsewhere  F03 - Unspecified dementia  G30 - Alzheimer disease |
| Inctontinence | N39 - Other disorders of urinary system  R15 - Faecal incontinence  R32 - Unspecified urinary incontinence |
| Infections | A40 - Streptococcal sepsis  A41 - Other sepsis  J15 - Bacterial pneumonia, not elsewhere classified  J16 - Pneumonia due to other infectious organisms, not elsewhere classified  J17 - Pneumonia in diseases classified elsewhere  J18 - Pneumonia, organism unspecified  J69 - Pneumonitis due to solids and liquids  M72.6 - Necrotizing fasciitis  M86 - Osteomyelitis  R57.2 - Septic shock  R65 - Systemic Inflammatory Response Syndrome [SIRS] |
| Other severe diseases | B37.1 - Pulmonary candidiasis  B37.7 - Candidal sepsis  D50 - Iron deficiency anaemia  D51 - Vitamin B12 deficiency anaemia  D52 - Folate deficiency anaemia  D53 - Other nutritional anaemias  D61 - Other aplastic anaemias  D62 - Acute posthaemorrhagic anaemia  D63 - Anaemia in chronic diseases classified elsewhere  D64 - Other anaemias  D72.8 - Other specified disorders of white blood cells  G63.2 - Diabetic polyneuropathy  K70 - Alcoholic liver disease  K72 - Hepatic failure, not elsewhere classified  K74 - Fibrosis and cirrhosis of liver  N17 - Acute renal failure  N18.4 - Chronic kidney disease, stage 4  N18.5 - Chronic kidney disease, stage 5  N99.0 - Postprocedural renal failure  R18 - Ascites  R34 - Anuria and oliguria  R60 - Oedema, not elsewhere classified  R77.0 - Abnormality of albumin  U69.00!* - hospital acquired pneumonia (valid until 2018)  Z99.2 - Dependence on renal dialysis |
| Mobility | G20 - Parkinson disease  G81 - Hemiplegia  G82 - Paraplegia and tetraplegia  G83 - Other paralytic syndromes  M24.5 - Contracture of joint  M62 - Other disorders of muscle  R26.3 - Immobility  R40 - Somnolence, stupor and coma  R46 - Symptoms and signs involving appearance and behaviour  S14 - Injury of nerves and spinal cord at neck level  S24 - Injury of nerves and spinal cord at thorax level  Z74.0 - Need for assistance due to reduced mobility  Z99.3 - Dependence on wheelchair |

* only applicable for German Modification

**S9 – Variable Importance and partial dependence plots statistically considering length of stay**

**S10 – Comparison of BART with alternative predictive models**

To assess the relative predictive performance of BART, we considered multiple alternative predictive models. These included logistic regression, random forest, and least absolute shrinkage and selection operator (LASSO). Models were fit using 10-fold cross validation based on data from 2014-2017. The tuning parameters of BART, random forest, and LASSO and their final values are shown in the table below. Based on the best predictive models identified through cross validation, we prospectively predicted PU cases in data from 2018.

| Model | Tuning parameter | Tuning parameter after cross validation |
| --- | --- | --- |
| *BART* | Number of trees {25,50,75,100} | 50 |
| *Random forest* | Number of variables to possibly split at in each node {1,2,..,10} | 3 |
| *LASSO* | Regularization parameter {0,0.005,…,1} | 0.035 |
